# Ventricular volume adjustment of brain regions depicts brain changes associated with HIV infection and aging better than intracranial volume adjustment

**DOI:** 10.3389/fneur.2025.1516168

**Published:** 2025-05-19

**Authors:** Benedictor Alexander Nguchu, Jing Zhao, Jiajun Fan, Yifei Han, Yu Lu, Han Jin, Yanming Wang, Hongjun Li, Peter Shaw

**Affiliations:** ^1^Oujiang Laboratory (Zhejiang Lab for Regenerative Medicine, Vision and Brain Health), Wenzhou Medical University, Wenzhou, Zhejiang, China; ^2^School of Ophthalmology and Optometry and Eye Hospital, Wenzhou Medical University, Wenzhou, Zhejiang, China; ^3^Department of Radiology, Beijing Youan Hospital, Capital Medical University, Beijing, China; ^4^School of Electronic Engineering, Tianjin University of Technology and Education, Tianjin, China Tianjin, China; ^5^Center for Biomedical Imaging, University of Science and Technology of China, Hefei, Anhui, China

**Keywords:** intracranial volume, lateral ventricle volume, basal ganglia, Hippocampus, thalamus, region volume proportion, human immunodeficiency virus, structural changes

## Abstract

**Introduction:**

While the adjustment of intracranial volume (ICV) is reported to have a significant influence in the outcomes of the analyses of brain structural measures, our study offers a paradigm shift, positing that adjusting for lateral ventricle (LV) inter-individual variability may reveal more atrophic patterns that might be overlooked in analyses without this adjustment,—and such LV-adjusted atrophic patterns may reduce discrepancies observed in earlier studies and better elucidate complex conditions associated with HIV, such as HAND.

**Methods:**

To test this hypothesis, we employed a number of adjustment strategies on MRI T1-image-derived data extracted using deep learning models and compared their ability to identify the presence and extent of HIV-specific atrophic patterns based on statistical measures and strength.

**Results:**

Our results show that both ICV adjustments may be effective to identify atrophic patterns associated with either aging or HIV in areas of the thalamus, basal ganglia, ventral DC and lateral ventricle, some of which may be overlooked without these adjustments. We also report that LV adjustmenst detect most atrophic patterns associated with HIV and HAND across multiple subcortical regions with more strong statistical strengths, especially the areas of the basal ganglia (putamen, pallidum, caudate nucleus), hippocampus, thalamus, ventral DC, basal forebrain, third ventricle, fourth ventricle, and inferior lateral ventricle. The analyses of LV-adjusted metrics also show that atrophic patterns observed in the hippocampus, thalamus and pallidum were strongly correlated with HAND(especially dysfunction in executive function) and clinical markers (i.e., CD4/CD8 ratio).

**Conclusion:**

We conclude that models that control for individual variability in intracranial and ventricular volumes have the potential to minimize discrepancies and variations in structural reports of HIV, improving the diagnostic power of identified patterns and fostering greater consistency across research studies. More importantly, adjusting for LV may not only detect atrophic patterns that could be overlooked in analyses performed without any adjustments, but the outcomes obtained from the adjustments may better explain HIV-associated conditions such as HAND and underlying immunological issues often observed in subjects with HIV treated with combination antiretroviral therapy, considering that the adjustments account for certain aspects of regional interaction.

## 1 Introduction

Recently, there is a growing concern regarding the diagnostic markers of neuropathology of *HIV* and conditions associated with *HIV* injury particularly H*IV*-associated neurocognitive disorders (*HAND*). While some studies show structural changes to be indicative of HIV pathology (1, 2), other studies present significant inconsistencies in the findings, suggesting a lack of reliability or reproducibility of the structural findings (3). While some of these studies report subcortical areas—particularly the thalamus and hippocampus (4, 5)—as the primary targets of *HIV*, other studies indicate that HIV effects are only evident in the putamen and caudate regions of the basal ganglia (1, 6). Others (3) even argue that structural changes such as those in volumes may not be sensitive enough for diagnosing HIV pathophysiology or conditions like *HAND* that can emerge after progressive attacks by HIV on the CNS (7). These inconsistencies and discrepancies across studies raise serious concerns in understanding the mechanisms of HIV pathology and related cognitive challenges.

In this study, we postulate that the inconsistencies and gaps observed across structural studies primarily stem from four main factors—study methods, participant characteristics, differential treatment practices, and comorbid conditions such as age or depression (8). Addressing these key factors may reduce such inconsistencies. We thus posit that (1) contrary to previous studies, structural changes are reliable biomarkers of HIV neuropathology and can effectively distinguish patterns linked to HIV pathology and HAND condition from normal patterns; (2) controlling for individual variability in intracranial or ventricular volume is necessary for the reliability and reproducibility (with high diagnostic power) of structural changes; (3) while both strategies for controlling individual variability can be promising, we stress that the strategy involving ventricular volume adjustment may reveal more distinct patterns associated with HIV than the strategy accounting for intracranial volume. We further hypothesize that controlling for ventricular volume is likely to enhance the diagnostic power and sensitivity of structural measures to HIV encephalitis—especially in the subcortical areas where individual variations influenced by ventricular volume are more prevalent due to the high spatial proximity and structural connectivity of these regions with the ventricular areas.

The bases of these hypotheses stem from the fact that both brain structures—the *ICV* and ventricular volumes—vary significantly across individuals even in the absence of neurological conditions. This fact has been documented extensively by Bigler at el. and Vojinovic et al. (9, 10). The condition known as hydrocephalus ex vacuo (11, 12) is largely associated with increased variability in overall intracranial size and ventricular volume, even among individuals of the same age. Increases in CSF-filled ventricular sizes without an increase in CSF pressure (13) are characteristic of this condition, which is linked to a compensatory mechanism for brain atrophy or decreasing brain tissue. Variations in genetic factors may differentially contribute to the onset of hydrocephalus ex vacuo (14). Other conditions occuring in some individuals at a young age—like intraventricular hemorrhage—can worsen later in life, causing severe damage and atrophy of brain tissue. This can lead to hydrocephalus ex vacuo and greater variability in ICV and ventricular space among individuals (15). On the basis of these facts, we bodly argue the analyses of structural changes in individuals with neurological conditions require accounting for variations in these structures for reliable inference. Aligning with our core concept is the study by Boedhoe et al. (16). The team employed the strategy of controlling for individual variability in intracranial volume in their structural analyses when investigating how neurological conditions such as ADHD (*N* = 2,271), ASD (*N* = 1,777), and OCD (*N* = 2,323) induce changes in the subcortical and surface areas of the brain. This strategy identified a greater volume reduction in the subcortical areas—particularly in the hippocampus—in children with ADHD compared to those with OCD (16), which links the neurodegeneration of subcortical areas to the pathogenesis of ADHD.

So far, despite the widely accepted fact that the lateral ventricle is the most variable structure of the brain across individuals (17), there are still limited studies accounting for this variability in structural analyses when attempting to detect potential patterns of brain atrophy. Bartos et al. (18) were the first to attempt to include this variable in structural analyses. They, in fact, used this variable to formulate a quantitative measure that distinguishes Alzheimer’s disease from normal subjects while improving sensitivity and specificity (18). Our study generalizes this concept and applies it not only to specific regions of subcortical structures, as used by Bartos et al., but to the entire subcortical regions. The reason for this generalization/concept expansion is that the volumes of the entire subcortical structures are typically reduced by neurological conditions such as aging and HIV, with the ventricular spaces generally expanding. Another reason for this generalization is that ventricular areas are structurally connected to these subcortical areas (19); thus, individual variability in the ventricular spaces directly affects these regions both structurally and spatially.

Other studies have attempted to address the problem of individual variability by proposing entirely new alternative approaches to conventional methods for quantifying changes in brain structures. These methods include fractal dimensionality (FD) and tensor-based morphometry (TBM) (20, 21). The methods are considered to address the problem by quantifying aspects of brain structures that are less sensitive to individual variability. For example, FD quantifies the complexity of brain structures and is recently recognized for its sensitivity to changes in morphometry (22, 23). In nueroimaging studies, FD utilizes the geometric complexity and self-similarity of the brain structure to describe and index its structural complexity. While this method was originally inspired to capture and describe the complexity of natural objects—such as the shapes of mountains, trees, and clouds—its early applications in neuroimaging proved effective, particularly in brain development (22, 24) and aging (21, 22, 25). In HIV/AIDS (26) FD has been used to study how closely this measure quantifies HIV-induced neural injury as compared to other measures, i.e., the cortical thickness and volumetric measures. It was found that FD in the caudate and parietal lobe was substantially reduced in a large sample of people living with HIV. The FD in four lobes (i.e., frontal, temporal, parietal, and occipital) and subcortical regions (caudate and hippocampus) were highly associated with cognition, suggesting better sensitivity of FD to structural modifications associated with cognition than conventional measures. While the FD results appeared to be more close to explaining cognitive functioning of the brian in this study, further investigation is still warranted to address the inconsistencies found in the FD findings for the two cohorts of the same study. While the large cohort showed FD changes only in the caudate and parietal lobe, the selected small cohort detected FD changes in the hippocampus and all four lobes. This was contrary to the volumetric measures controlled for intracranial volume variability, which appeared consistent for both small and large cohorts. Furthermore, there is an ongoing concern that FD measurement is significantly affected by the magnetic field strengths (1.5 T, 3 T, and 7 T) of imaging, with higher field strength like 7 T showing higher FD values than the lower field strengths (1.5 T and 3 T) (27). This phenomenon highlights that FD measurements consider fine details of the image that may be highly visible in higher field strengths of magnetization like 7 T, and as such, its estimation is likely to be affected by MRI noise in lower field strengths. This also brings awareness that FD measures different aspects of structural changes compared to conventional methods, and therefore there is still a need of additional metrics (including volumetric measures, cortical thickness, and gyrification) to assess and index different aspects of atrophy unxplained/unrelated to FD (21) while accounting for other confounding factors.

On these grounds, we conduct our study with a high degree of confidence and the assumption that controlling for these global parameters—the ICV and ventricular volumes, which often induce high individual variability in volumetric analyses—may improve the margin of group differences between normal brains and those with HIV pathology or other conditions associated with HIV injury, possibly unraveling new findings that were obscured by the high degree of individual variations. To carry out our study, we have collected data from 145 individuals, wherein 45 were healthy subjects and 100 were infected with *HIV-1*. We utilized deep learning models to extract volumes of the brain regions from *T1*-weighted images, followed by normalization of these volumes. The resulting proxy measures of structural changes were statistically compared to test their strength in distinguishing healthy individuals from those infected with *HIV*, as well as from those with *HAND*. The margins of differences revealed by these parameters controlled for *ICV* and ventricular volumes were further compared to the margins of differences reported by the data without any controlling strategy. The findings obtained from this study not only add knowledge to the current literature but also offer a degree of confidence regarding the usefulness of structural analyses and the conventional methods in studying brain changes or mechanisms underlying the complex conditions affecting the central nervous system.

## 2 Methods

### 2.1 Participants

One hundred and forty five participants were recruited at Beijing You An Hospital, the Capital Hospital, after obtaining written informed consent from each participant. The ethical committee of the Capital Medical University, the University of Science and Technology of China, and Wenzhou Medical University approved this study. The experiments and protocols carried out in this study complied with the code of ethics of the World Medical Association (Declaration of Helsinki) for human experiments. Individuals with signs of neurological disorders, brain injury, brain lesions, cerebral atrophy, or illicit drugs and alcohol were excluded from participating. Of 145, 100 individuals had *HIV-1*, and were receiving antiretroviral therapy, i.e., the combination of tenofovir (*TDF*) + lamivudine (*3TC*) + efavirenz (*EFV*). Blood assays were performed to determine the number of copies of HIV-1 per milliliter of blood (copies/mL), the number of *CD4-T* cells per microliter of blood (cells/μL), and the number of *CD8 T* cells per microliter of blood (cells/μL). For each patient with *HIV-1*, we conducted a cognitive assessment using a battery of neuropsychological tests to evaluate the performance of their cognitive domains and determine if they are impaired.

#### 2.1.1 Neuropsychological testing (procedures and criteria)

Testing for the cognitive performance was based on self-reports and a battery of neuropsychological assessment tests. Briefly, the testing was performed for six cognitive domains, and patients at risk of cognitive impairment (*HAND*) were identified based on Frascati rating scales of 2007 to define *HAND* (28). The six cognitive domains examined include (1) attention and working memory tested using the Paced Auditory Serial Addition Test (*PASAT*), Continuous Performance Test Identical Pairs (*CPT-IP*), and the Wechsler Memory Scale-III (*WMS-III*); (2)Verbal and language tested using the Category Fluency and Animal Naming Tests; (3) Motor function tested using the Grooved Pegboard Test; (4) abstract and executive function tested using the Wisconsin Card Sorting Test-64 (*WCST-64*) Test; (5) learning and recall tested using the Hopkins Verbal Learning Test-Revised (*HVLT-R*) and the Brief Visuospatial Memory Test-Revised (*BVMT-R*) Tests; and (6) information processing speed tested using the Trail-Making Test Part A. Standardization of raw scores for each test was performed to obtain T-scores with demographic adjustments. The final composite T-score of cognitive domain tested using multiple tests was obtained by averaging the T-scores from all tests performed in the domain. A patient who demonstrated impairment in at least two cognitive domains but maintained normal functioning in daily activities was classified as having asymptomatic cognitive impairment (*ANI*, an early stage of *HAND*).

#### 2.1.2 MRI neuroimaging

We scanned the brains of our participants at Beijing You An Hospital, the Capital Hospital using a *3 T MRI* scanner equipped with a 32-channel head coil (Allegra, Siemens Medical System, Erlangen, Germany). The images were obtained using *3D-T1*-weighted image sequence (MPRAGE), set at the following configurations: *TR/TE* = 1,900 ms/2.52 ms, inversion time = 900 ms, flip angle = 9°, field of view (*FOV*) = 250 mm^2^ × 250 mm^2^, matrix size = 246 × 256, slice thickness = 1 mm, and voxel size = 1 × 1 × 1 mm^3^.

#### 2.1.3 Imaging data pre-processing and volumetric estimates

Data preprocessing was performed based on the pipelines described previously for anatomical data. Briefly, the images of anatomical structures were segmented into three main tissues—*CSF*, white matter, and gray matter tissues. Volumetric estimates of brain regions in native space were then obtained using deep learning models integrated into a fully automated pipeline offered by VolBrain.^1^ We used the pipeline described by Coupé et al. (29) and de Senneville et al. (30), which employs AssemblyNet for cortical parcellation, intracranial cavity, and brain tissue segmentation (29, 30). We used the pipeline described by Coupé et al. (29), which uses non-linear warping and multi-atlas label fusion technology for the segmentation of subcortical structures (31). Both gray matter and white matter volumes for the cortical and subcortical structures were measured. We next obtained three sets of volumetric estimates. The first set *Ȿ_1_* contained the raw volumetric estimates that were not controlled for either intracranial volume or ventricular volume. The second set *Ȿ_2_* contained volumetric estimates controlled/accounting for intracranial volume variability using normalization or covariation. The following formula was applied for normalization by *ICV*: *V_E_ = V_R_/ICV*, where *ICV* is the intracranial volume, *V_R_* is regional volume, and V_E_ is estimated regional volume fraction accounting for *ICV* variability. The third set Ȿ_3_ contained volumetric estimates accounting for ventricular volume variability. The estimates were obtained by normalization, volume proportion, or covariation. For normalization by *LV*, the following formula was applied: *V_E_ = V_R_/LV*, where *LV* is the ventricular volume, *V_R_* is regional volume, and *V_E_* is estimated regional volume fraction accounting for ventricular variability. For volume proportions accounting for ventricular volume, the following formula was applied: region_τ_ volume / (region_τ_ volume + Lateral ventricle volume).

#### 2.1.4 Statistical analysis

We performed statistical computations on R v4.2.0, an environment for statistical computing (32). We first analyzed the effects of Age, *HIV*, and *HAND* status on the three sets of volumetric estimates (involving raw volumetric data, *ICV*-adjusted data, and LV-adjusted data). Next, we conducted *post-hoc* analyses to identify trends and the magnitude of statistical differences between groups and subgroups. The diagnostic power of the three sets of volumetric estimates was evaluated based on statistical values such as *p*-values, *F*-values, and effect sizes. We further performed correlation analyses to determine which of the three sets of volumetric estimates are strongly associated with blood test and neuropsychological test reports.

#### 2.1.5 Receiver operating characteristic analysis

To evaluate the performance of each technique in distinguishing binary classes or groups, particularly HAND vs. non-HAND, we conducted a receiver operating characteristic (ROC) analysis. We specifically focused on the patterns or regions that were significantly different between the groups. We plotted the ROC curves and observed the diagnostic power of each method’s derived metrics by assessing their area under the curve (AUC) and their statistical *p*-values.

## 3 Results

### 3.1 Absolute volume analyses without adjusting for individual variability

**HIV effects**: The benchmark analysis, which involved examining the volumes of brain regions without adjusting for individual variability, identified HIV effects on the volumes of the lateral ventricle (LV) (Table 1). These HIV effects were seen in both the right and left sides of the *LV*, and they were reflected in the overall *LV* volume. In essence, subjects with HIV demonstrated a greater increase in LV volume compared to those who were healthy (Pairwise comparisons; *HC-HIV*: overall CE = −3.4989, *p* < 0.0025, eff.size = −0.5654, right CE = −1.4916, *p* < 0.0066, eff.size = −0.5058, left CE = −2.0078, *p* < 0.00242, eff.size = −0.5669). These observations suggest that the infection of CNS by *HIV* may be causing the expansion/enlargement of lateral ventricle spaces. Further *post-hoc* analysis indicated that HIV subjects diagnosed with HAND demonstrated greater atrophy than HIV subjects without HAND when both groups were compared with HC (HAND+: overall LV, CE = 4.4307, *p* < 0.0056, eff.size = 0.7168; HAND-: CE = 2.9497, *p* < 0.05, eff.size = 0.4771; Supplementary Table S1). Such observations were not only identified in the overall *LV* volume but were also observed on both sides of the *LV* [(HAND+) – HC: right CE = 1.9333, *p* < 0.0129, eff.size = 0.6563, left CE = 2.4967, *p* < 0.0066, eff.size = 0.7051; (HAND-) – HC: right CE = 1.2313, *p* < 0.1134, eff.size = 0.4180, left CE = 1.7198, *p* < 0.0483, eff.size = 0.4857].

**Table 1:** Atrophic patterns identified from raw volumetric measures and ICV-adjustments through covariation

Brain structures	HIV	HC	Young	Old	Age effect				HIV effect			
					F	*p*	Contrast estimate	Effect size	F	*p*	Contrast estimate	Effect size
Absolute volumes (mm^3^)												
Pallidum	3±0.28	3.03±0.32	3.08±0.25	2.96±0.31	7.4713	**0.0071**	0.1348	0.4725	1.0895	0.2984	0.0547	0.1915
Putamen	9.67±0.94	9.47±0.98	9.86±0.84	9.41±0.98	7.3159	**0.0077**	0.4346	0.4675	0.4576	0.4999	−0.1154	−0.1241
Lateral Ventricle (LV)	16.2±6.97	13.4±4.89	13.7±5.15	16.5±7.13	10.0538	**0.0019**	−3.3917	−0.5481	9.4926	**0.0025**	−3.4989	−0.5654
R.Pallidum	1.49±0.14	1.5±0.16	1.52±0.13	1.47±0.15	5.1415	**0.0249**	0.0554	0.3919	0.7623	0.3841	0.0227	0.1602
R.Putamen	4.85±0.47	4.76±0.5	4.96±0.42	4.72±0.5	8.5213	**0.0041**	0.2360	0.5046	0.2521	0.6164	−0.0431	−0.0921
R.LV	7.48±3.15	6.32±2.82	6.28±2.37	7.73±3.41	11.1743	**0.0011**	−1.7038	−0.5778	7.5982	**0.0066**	−1.4916	−0.5058
L.Pallidum	1.52±0.16	1.53±0.18	1.56±0.13	1.49±0.18	7.5686	**0.0067**	0.0779	0.4755	1.1991	0.2754	0.0329	0.2009
L.Putamen	4.81±0.48	4.7±0.49	4.9±0.44	4.69±0.5	6.0183	**0.0154**	0.2005	0.4240	0.6459	0.4230	−0.0697	−0.1475
L.LV	8.71±3.99	7.03±2.67	7.4±2.97	8.74±4.07	7.6075	**0.0066**	−1.6886	−0.4767	9.5421	**0.0024**	−2.0078	−0.5669
ICV adjustments through covariation												
Pallidum	3±0.03	3.06±0.04	3.09±0.04	2.97±0.03	6.7723	**0.0103**	0.1252	0.4508	1.3001	0.2562	0.0581	0.2093
Putamen	9.66±0.09	9.56±0.13	9.81±0.12	9.42±0.1	6.6246	**0.0111**	0.3901	0.4459	0.3843	0.5363	−0.0996	−0.1138
Thalamus	12.99±0.11	13.38±0.16	13.28±0.15	13.09±0.12	1.0838	1.0838	0.1897	0.1804	3.9010	**0.05**	0.3812	0.3625
LV	16.09±0.61	12.68±0.92	12.57±0.83	16.2±0.67	12.3366	**0.0006**	−3.6294	−0.6085	9.7248	**0.0022**	−3.4142	−0.5724
R.Pallidum	1.49±0.01	1.51±0.02	1.52±0.02	1.47±0.02	4.5394	**0.0349**	0.0511	0.3691	0.9100	0.3418	0.0242	0.1751
R.Putamen	4.85±0.04	4.82±0.07	4.94±0.06	4.73±0.05	7.8521	**0.0058**	0.2133	0.4855	0.1886	0.6648	−0.0350	−0.0797
R.Thalamus	6.51±0.06	6.74±0.09	6.67±0.08	6.58±0.07	0.7187	0.3980	0.0857	0.1469	4.4973	**0.0357**	0.2271	0.3893
R.LV	7.43±0.29	5.99±0.43	5.79±0.39	7.62±0.32	14.0947	**0.0003**	−1.8299	−0.6504	7.8479	**0.0058**	−1.4467	−0.5142
L.Pallidum	1.52±0.02	1.55±0.02	1.57±0.02	1.5±0.02	6.8786	**0.0097**	0.0727	0.4544	1.4036	0.2382	0.0348	0.2175
L.Putamen	4.81±0.05	4.75±0.07	4.87±0.06	4.69±0.05	5.3203	**0.0226**	0.1787	0.3996	0.5699	0.4516	−0.0620	−0.1386
L.Thalamus	6.48±0.05	6.64±0.08	6.61±0.07	6.51±0.06	1.3805	0.2420	0.1028	0.2036	2.7774	0.0979	0.1545	0.3059
L.LV	8.66±0.35	6.69±0.53	6.78±0.48	8.58±0.39	9.0171	**0.0032**	−1.8003	−0.5202	9.5995	**0.0024**	−1.9681	−0.5687
LV adjustments through covariation												
Pallidum	3±0.3	3.06±0.05	3.1±0.04	2.96±0.03	7.2345	**0.0080**	0.1378	0.4814	1.1321	0.2892	0.0578	0.2018
Putamen	9.68±0.1	9.54±0.15	9.82±0.13	9.4±0.11	6.2646	**0.0135**	0.4177	0.4480	0.5638	0.4540	−0.1328	−0.1424
R.pallidum	1.49±0.01	1.51±0.02	1.52±0.02	1.47±0.02	4.2796	**0.0404**	0.0525	0.3703	0.5339	0.4662	0.0197	0.1386
R.Putamen	4.86±0.05	4.81±0.07	4.95±0.07	4.72±0.05	7.9833	**0.0054**	0.2374	0.5057	0.2189	0.6406	−0.0417	−0.0887
L.pallidum	1.52±0.02	1.55±0.03	1.58±0.02	1.49±0.02	8.0515	**0.0052**	0.0834	0.5079	1.5330	0.2178	0.0385	0.2348
L.Putamen	4.82±0.05	4.73±0.07	4.87±0.07	4.69±0.05	4.6238	**0.0333**	0.1822	0.3849	0.9744	0.3253	−0.0886	−0.1872

The bold values indicate the *P*-values that were statistically significant, at the threshold of *P* < 0.05.

**Age-related effects**: This benchmark analysis also identified age-related effects in our data. There were widespread age-related effects in the LV and structures of the basal ganglia—particularly the putamen and pallidum. Both the right and left sides of these brain structures— showed volume changes due to aging (see Table 1). Those who were older had larger LV volumes compared to younger subjects (young – old: LV, overall CE = −3.3917, *p* = 0.0019, eff.size = −0.5481). The volumes of the basal ganglia structures were significantly reduced in older subjects compared to younger ones (young – old: pallidum, overall CE = 0.1348, *p* = 0.0071, eff.size = 0.4725; putamen, overall CE = 0.4346, *p* = 0.0077, eff.size = 0.4675). These results demonstrate the independent effects of age on the brain, with structures like the basal ganglia and *LV* revealing more pronounced effects even in the absence of variability-controlling strategies.

### 3.2 Absolute volume analyses with variable adjustment

(a) Adjusting ICV as a covariate.

(1) For HIV effects: Adjusting ICV as a covariate in the model (Table 1) identified the thalamus as another region whose volume demonstrated changes due to HIV, in addition to the lateral ventricle. More pronounced HIV-related effects were identified in the right side of the thalamus (*F* = 4.4973, *p* = 0.0357), while fewer effects were identified in the left side (*F* = 2.7774, *p* = 0.0979). From pairwise comparison analyses, we learned that the effects of HIV on the thalamus resulted in a greater reduction in thalamic volume for those with HIV compared to normal subjects (*HC – HIV*: overall CE = 0.3812, *p* = 0.0050, eff.size = 0.3625; right CE = 0.2271, *p* = 0.0357, eff.size = 0.3893; left CE = 0.1545, *p* = 0.0979, eff.size = 0.3059). Of importance to note is that, including *ICV* as a covariate, bolstered the statistical power of the *LV* outcomes. The *p*-values and effect sizes of the LV outcomes improved and became more robust statistically (HC – HIV: overall CE = −3.4142, *p* = 0.0022, eff. Size = −0.5724; right CE = −1.4467, *p* = 0.0058, eff. Size = −0.5142; left CE = −1.9681, *p* < 0.0024, eff. Size = −0.5687) compared to the outcomes reported in the benchmark analysis (i.e., without any adjustments). These findings underscore the importance of adjusting for *ICV* in revealing more patterns of *HIV* pathology.
(2) For HAND effects: Adjusting for ICV by covariation improved the statistical power for the *post-hoc* analysis of HAND+ and HAND- vs. HC (Supplementary Table S1). This adjustment resulted in larger effect sizes and smaller p-values for the outcomes of comparisons between subjects with and without HAND against normal subjects, particularly those involving changes in LV [(HAND+) – HC: overall CE = 4.3332, *p* = 0.0048, eff.size = 0.7274; right CE = 1.8816, *p* = 0.0108, eff.size = 0.6697; left CE = 2.4509, *p* = 0.0063, eff.size = 0.7085; (HAND-) – HC: overall CE = 2.8729, *p* = 0.0506, eff.size = 0.4823; right CE = 1.1905, *p* = 0.1060, eff.size = 0.4237; left CE = 1.6837, *p* = 0.04768, eff.size = 0.4867], contrast with the results obtained without adjusting for *ICV*. It is important to note that, while this adjustment of ICV revealed significant effects of HIV on thalamic volume, we did not detect patterns of changes in this region that could distinguish HAND+ from HAND- or differentiate both HAND- and HAND+ subjects from normal subjects.
(3) For age-related effects: The inclusion of ICV as a covariate did not diminish or obscure the age-related effects on the brain identified in the benchmark analysis (i.e., analyses performed without any adjustment). Nevertheless, this inclusion boosted the statistical power of the outcomes. The statistical values for age-related changes in LV improved to (*F* = 12.336635, *p* = 0.0006) from (*F* = 10.05378, *p* = 0.00187), to (*F* = 14.0947, *p* = 0.0002) from (*F* = 11.1742, *p* = 0.0011), and to (*F* = 9.0171, *p* = 0.0032) from (*F* = 7.6075, *p* = 0.0066) for the overall *LV* volume, right *LV* volume, and left *LV* volume, respectively. The age-related changes identified in the benchmark analysis on basal ganglia structures (pallidum and putamen) were still detectable in this analysis, adjusting for ICV, with similar statistical strengths (see Table 1).

(b) Adjusting LV as a covariate.

(1) For age-related effects: The inclusion of *LV* as a covariate did not affect the first age-related results obtained without any adjustment, except for the results associated with *LV* itself (Table 1). The basal ganglia structures—both the pallidum and putamen—still showed patterns of volumetric changes associated with age. There were slight, noticeable improvements in the statistical parameters for the changes identified by this adjustment. Noticeably, the statistical values of the left pallidum improved from (*F* = 7.5686, *p* = 0.0067, CE = 0.0779, eff. Size = 0.4755) at benchmark analysis to (*F* = 8.0515, *p* = 0.0052, CE = 0.0834, eff. Size = 0.5079) after LV adjustment.
(2) For HIV and HAND effects: This adjustment (LV covariation) significantly altered the first results associated with *HIV* pathology identified in the benchmark analysis (analysis without any adjustment). This was expected because earlier results in the benchmark analysis implicated only lateral ventricle volume in HIV-related pathology. Thus, incorporating *LV* in the model automatically offset the results involving *LV*. However, this adjustment signaled the presence of new patterns of volumetric change (but they did not pass the statistical threshold) in the right thalamus (*F* = 3.0379, *p* = 0.0836, CE = 0.2172, eff.size = 0.3306) and left inferior *LV* (*F* = 3.4004, *p* = 0.0673, CE = 0.0608, eff.size = 0.3498). This strategy did not identify any patterns related to HAND either. Only the basal forebrain demonstrated a minor trend of HAND-related alterations, but they did not pass the statistical threshold [(HAND+) – HC: overall CE = 0.0308, *p* = 0.0687, eff.size = 0.5387].

### 3.3 Volume analyses with normalized data

(a) Normalizing data with ICV.

(1) ICV-normalization for HIV and HAND effects: Adjusting for *ICV* by normalizing regional volumes with global *ICV* enhanced the identification of patterns associated with both *HIV* pathology and age. With this strategy, two additional regions—the pallidum and thalamus—alongside the lateral ventricle, were identified to exhibit volumetric alterations as a result of HIV infection (see Table 2). These alterations were seen on both sides of the regions. The observations were supplemented by stronger statistical measures, reinforcing the reliability of the observed changes. From *post-hoc* analyses of ICV-normalized data, we learned that both subjects with and without *HAND+* demonstrated a significant reduction in thalamic volume and a significant increase in lateral ventricle volume compared to normal subjects (Supplementary Table S2). We also detected significant patterns of volumetric changes linked to *HAND*. Those diagnosed with HAND(HAND+) had a greater reduction in thalamic volume and a greater increase in LV volume than HAND- individuals when compared to normal subjects ((HAND+) -HC [overall thalamus, CE = −0.00042, *p* = 0.0071, eff.size = −0.7000; overall LV, CE = 0.0029, *p* = 0.0054, eff.size = 0.7201], (HAND-)-HC[overall thalamus, CE = −0.00036, *p* = 0.0101, eff.size = −0.5945; overall LV, CE = 0.0019, *p* = 0.0544, eff.size = 0.4766]). These findings suggest that changes in the normalized data, particularly in the thalamus and lateral ventricle, may better predict HAND progression.
(2) ICV-normalization for age-related effects: Normalizing data by ICV improved the detection of patterns associated with the aging process. In addition to the basal ganglia structures (i.e., pallidum and putamen) and lateral ventricle, which were also detected in the first strategy (benchmark analysis), two more brain regions—the thalamus and ventral *DC*—were identified to exhibit alterations related to age. These patterns were supported by strong statistical power, suggesting a high degree of fidelity in our results.

(b) Normalizing data with LV.

(1) LV-normalization for HIV and HAND effects: Table 3 shows the results obtained by normalizing regional volumes by lateral ventricle volume. Our data indicate that normalization by *LV* yielded high diagnostic power, as evidenced by statistical measures. Several brain regions displayed patterns that indicate modifications linked to *HIV*. The most significant patterns (*p* < 0.00*) were identified bilaterally across the structures of the hippocampus, basal ganglia (pallidum and putamen), thalamus, ventral *DC*, third ventricle, and fourth ventricle. *Post-hoc* analyses demonstrated a high degree of accuracy in diagnosing *HAND* from *LV*-normalized data (Supplementary Table S3). HAND subjects showed a greater contrast when compared to normal subjects, while non-HAND subjects showed minimal contrast. Such observations were particularly identified and pronounced in the hippocampus, basal ganglia (pallidum and putamen), thalamus, ventral *DC*, and fourth ventricle. These results imply that normalization techniques based on *LV* can reveal brain changes that may not be detected through alternative methodologies.
(2) LV-normalization for age-related effects: This normalization strategy identified several brain regions exhibiting age-related changes. The patterns of changes related to aging were pronounced (*p* < 0.000*) within the basal forebrain, caudate nucleus, pallidum, and putamen. Less prominent (*p* < 0.00*) patterns were detected in the hippocampus, thalamus, ventral *DC*, and third ventricle. These observations suggest that data normalization by *LV* allows for the identification of more patterns associated with both age and *HIV* pathology than using alternative strategies.

**Table 2:** Atrophic patterns identified through ICV-adjustments via normalization.

Brain structures	HIV	HC	Young	Old	Age effect				HIV effect			
					F	*p*	Contrast estimate	Effect size	F	*p*	Contrast estimate	Effect size
ICV adjustments through normalization												
Gray Matter (GM) normalized	0.5106 ± 0.0217	0.5079 ± 0.0265	0.5172 ± 0.0252	0.5043 ± 0.0202	10.8518	**0.0013**	0.0128	0.5694	0.0034	0.9534	−0.0002	−0.0107
Subcortical Gray Matter normalized	0.0316 ± 0.0017	0.0319 ± 0.0014	0.0321 ± 0.0016	0.0314 ± 0.0016	8.0249	**0.0053**	0.0008	0.4896	2.0706	0.1524	0.0004	0.2641
Cortical Gray Matter normalized	0.402 ± 0.0183	0.401 ± 0.022	0.407 ± 0.0213	0.3977 ± 0.0171	8.0841	**0.0051**	0.0094	0.4915	0.0564	0.8126	0.0008	0.0436
Cerebellar Gray Matter normalized	0.0771 ± 0.0065	0.0751 ± 0.0067	0.0781 ± 0.0065	0.0752 ± 0.0064	5.8482	**0.0169**	0.0027	0.418	1.5953	0.2087	−0.0015	−0.2318
Cerebro Spinal Fluid (CSF) normalized	0.1124 ± 0.0241	0.1078 ± 0.0289	0.1064 ± 0.0277	0.1142 ± 0.0237	4.1223	**0.0442**	−0.0089	−0.3509	1.8515	0.1758	−0.0063	−0.2497
Brain (WM + GM) normalized	0.8738 ± 0.0239	0.8781 ± 0.0287	0.8797 ± 0.0276	0.8718 ± 0.0235	4.1947	**0.0424**	0.0089	0.354	1.6925	0.1954	0.006	0.2387
Pallidum normalized	0.0021 ± 0.0002	0.0021 ± 0.0002	0.0021 ± 0.0002	0.002 ± 0.0002	11.4428	**0.0009**	0.0001	0.5847	4.4804	**0.0361**	0.00007	0.3884
Putamen normalized	0.0066 ± 0.0006	0.0066 ± 0.0005	0.0068 ± 0.0005	0.0065 ± 0.0006	11.5349	**0.0009**	0.0003	0.587	0.0031	0.9559	0.00001	0.0102
Thalamus normalized	0.0089 ± 0.0006	0.0092 ± 0.0007	0.0091 ± 0.0005	0.0089 ± 0.0007	5.3371	**0.0223**	0.0002	0.3993	11.9885	**0.0007**	0.0004	0.6354
Ventral DC normalized	0.0068 ± 0.0004	0.0069 ± 0.0005	0.0069 ± 0.0004	0.0068 ± 0.0004	3.8738	**0.05**	0.0001	0.3402	0.8924	0.3465	0.00008	0.1734
LV normalized	0.011 ± 0.0046	0.0092 ± 0.0031	0.0094 ± 0.0033	0.0113 ± 0.0047	10.8199	**0.0013**	−0.0023	−0.5686	9.5196	**0.0025**	−0.0023	−0.5662
R.Pallidum normalized	0.001 ± 0.0001	0.001 ± 0.0001	0.001 ± 0.0001	0.001 ± 0.0001	7.1536	**0.0084**	0.00004	0.4623	3.2345	0.0743	0.00003	0.33
R.Putamen normalized	0.0033 ± 0.0003	0.0033 ± 0.0003	0.0034 ± 0.0003	0.0032 ± 0.0003	13.3045	**0.0004**	0.0002	0.6305	0.0608	0.8056	0.00001	0.0452
R.Thalamus normalized	0.0045 ± 0.0003	0.0047 ± 0.0004	0.0046 ± 0.0003	0.0045 ± 0.0004	4.1672	**0.0431**	0.0001	0.3528	12.5551	**0.0005**	0.0002	0.6502
R.Ventral DC normalized	0.0034 ± 0.0002	0.0034 ± 0.0002	0.0034 ± 0.0002	0.0034 ± 0.0002	3.9483	**0.0489**	0.0001	0.3435	1.2639	0.2629	0.00004	0.2063
R.LV normalized	0.0051 ± 0.0021	0.004 ± 0.0018	0.0043 ± 0.0015	0.0053 ± 0.0022	11.9315	**0.0007**	−0.0011	−0.5971	7.5968	**0.0066**	−0.001	−0.5058
L.Pallidum normalized	0.001 ± 0.0001	0.0011 ± 0.0001	0.0011 ± 0.0001	0.001 ± 0.0001	12.1959	**0.0006**	0.0001	0.6036	4.5769	**0.0342**	0.00004	0.3926
L.Putamen normalized	0.0033 ± 0.0003	0.0033 ± 0.0003	0.0034 ± 0.0003	0.0032 ± 0.0003	9.4884	**0.0025**	0.0002	0.5324	0.0097	0.9216	−0.00001	−0.0181
L.Thalamus normalized	0.0044 ± 0.0003	0.0046 ± 0.0003	0.0045 ± 0.0003	0.0045 ± 0.0003	5.5715	**0.0196**	0.0001	0.408	9.2239	**0.0029**	0.0002	0.5573
L.Ventral DC normalized	0.0034 ± 0.0002	0.0034 ± 0.0003	0.0035 ± 0.0002	0.0034 ± 0.0002	3.5104	0.0631	0.0001	0.3238	0.5074	0.4775	0.00003	0.1307
L.LV normalized	0.0059 ± 0.0026	0.0048 ± 0.0017	0.0051 ± 0.0019	0.006 ± 0.0027	8.171	**0.0049**	−0.0012	−0.4941	9.4872	**0.0025**	−0.0013	−0.5652

The bold values indicate the *P*-values that were statistically significant, at the threshold of *P* < 0.05.

**Table 3:** Atrophic patterns identified through LV-adjustments via normalization.

Brain structures	HIV	HC	Young	Old	Age effect				HIV effect			
					F	*p*	Contrast estimate	Effect size	F	*p*	Contrast estimate	Effect size
LV-adjustments by normalization												
BasalForebrain / LV	0.06 ± 0.03	0.07 ± 0.03	0.08 ± 0.03	0.06 ± 0.02	12.1774	**0.0006**	0.0159	0.6032	5.4002	**0.0216**	0.0112	0.4264
Caudate / LV	0.56 ± 0.24	0.64 ± 0.24	0.66 ± 0.26	0.54 ± 0.22	11.7014	**0.0008**	0.1362	0.5913	5.8288	**0.0171**	0.1021	0.443
Hippo / LV	0.64 ± 0.27	0.75 ± 0.31	0.74 ± 0.31	0.63 ± 0.26	7.3631	**0.0075**	0.1294	0.469	7.9323	**0.0056**	0.1426	0.5168
Pallidum / LV	0.22 ± 0.09	0.26 ± 0.11	0.26 ± 0.11	0.21 ± 0.09	13.0516	**0.0004**	0.0592	0.6244	10.4927	**0.0015**	0.0564	0.5944
Putamen / LV	0.71 ± 0.3	0.82 ± 0.35	0.84 ± 0.35	0.67 ± 0.28	12.8604	**0.0005**	0.1900	0.6199	7.0874	**0.0087**	0.1497	0.4885
Thalamus / LV	0.95 ± 0.4	1.14 ± 0.45	1.12 ± 0.44	0.94 ± 0.4	10.0662	**0.0019**	0.2224	0.5484	10.1306	**0.0018**	0.2368	0.5841
Ventral_DC / LV	0.72 ± 0.29	0.85 ± 0.36	0.85 ± 0.35	0.7 ± 0.29	10.7328	**0.0013**	0.1730	0.5663	8.5388	**0.0041**	0.1638	0.5362
INF_LV / LV	0.03 ± 0.02	0.04 ± 0.05	0.04 ± 0.04	0.03 ± 0.01	6.6707	**0.0108**	0.0130	0.4464	5.9604	**0.0159**	0.0130	0.448
Third.V / LV	0.09 ± 0.03	0.1 ± 0.04	0.1 ± 0.03	0.09 ± 0.03	8.1351	**0.0050**	0.0143	0.4930	8.7469	**0.0036**	0.0158	0.5427
Fourth.V / LV	0.13 ± 0.05	0.15 ± 0.07	0.15 ± 0.07	0.13 ± 0.05	6.1649	**0.0142**	0.0247	0.4292	7.5254	**0.0069**	0.0290	0.5034
Ex.CSF / LV	10.11 ± 3.8	11.57 ± 5.4	11.27 ± 4.97	10.07 ± 3.89	4.0729	**0.0455**	1.5068	0.3488	4.9031	**0.0284**	1.7552	0.4063
R.BasalForebrain / R.LV	0.03 ± 0.01	0.04 ± 0.01	0.04 ± 0.01	0.03 ± 0.01	13.0807	**0.0004**	0.0081	0.6251	5.2645	**0.0233**	0.0055	0.4211
R.Caudate / R.LV	0.28 ± 0.12	0.32 ± 0.12	0.33 ± 0.13	0.27 ± 0.11	11.2132	**0.0010**	0.0664	0.5788	6.019	**0.0154**	0.0517	0.4502
R.Hippo / R.LV	0.32 ± 0.13	0.39 ± 0.16	0.38 ± 0.16	0.32 ± 0.13	7.5388	**0.0068**	0.0662	0.4746	8.2965	**0.0046**	0.0737	0.5286
R.Pallidum / R.LV	0.11 ± 0.05	0.13 ± 0.06	0.13 ± 0.05	0.11 ± 0.05	11.1337	**0.0011**	0.0279	0.5767	9.5724	**0.0024**	0.0274	0.5678
R.Putamen / R.LV	0.35 ± 0.15	0.41 ± 0.17	0.42 ± 0.17	0.34 ± 0.14	13.4671	**0.0003**	0.0966	0.6343	7.3829	**0.0074**	0.0759	0.4986
R.Thalamus / R.LV	0.48 ± 0.2	0.57 ± 0.22	0.56 ± 0.22	0.47 ± 0.2	10.2845	**0.0017**	0.1113	0.5543	10.7304	**0.0013**	0.1207	0.6011
R.Ventral_DC / R.LV	0.36 ± 0.15	0.42 ± 0.18	0.42 ± 0.17	0.35 ± 0.14	10.7217	**0.0013**	0.0858	0.566	8.6297	**0.0039**	0.0817	0.5391
R.INF_LV / R.LV	0.02 ± 0.01	0.02 ± 0.02	0.02 ± 0.02	0.02 ± 0.01	5.5754	**0.0196**	0.0064	0.4081	3.4789	**0.0643**	0.0054	0.3423
L.BasalForebrain / L.LV	0.03 ± 0.01	0.04 ± 0.02	0.04 ± 0.02	0.03 ± 0.01	10.8486	**0.0013**	0.0079	0.5693	5.0572	**0.0261**	0.0057	0.4127
L.Caudate / L.LV	0.28 ± 0.12	0.32 ± 0.12	0.33 ± 0.13	0.27 ± 0.11	12.0971	**0.0007**	0.0698	0.6012	5.6080	**0.0193**	0.0504	0.4346
L.Hippo / L.LV	0.31 ± 0.13	0.37 ± 0.15	0.36 ± 0.15	0.31 ± 0.13	7.1672	**0.0083**	0.0633	0.4627	7.5475	**0.0068**	0.0690	0.5041
L.Pallidum / L.LV	0.11 ± 0.05	0.13 ± 0.06	0.13 ± 0.05	0.11 ± 0.04	14.8093	**0.0002**	0.0312	0.6652	11.3118	**0.0010**	0.0290	0.6172
L.Putamen / L.LV	0.35 ± 0.15	0.41 ± 0.18	0.42 ± 0.17	0.34 ± 0.14	12.2878	**0.0006**	0.0935	0.6059	6.8269	**0.0100**	0.0740	0.4795
L.Thalamus / L.LV	0.47 ± 0.2	0.57 ± 0.23	0.56 ± 0.22	0.47 ± 0.2	9.772	**0.0022**	0.1110	0.5403	9.4947	**0.0025**	0.1162	0.5654
L.Ventral_DC / L.LV	0.36 ± 0.15	0.43 ± 0.18	0.43 ± 0.17	0.35 ± 0.15	10.714	**0.0013**	0.0872	0.5658	8.3982	**0.0044**	0.0820	0.5318
L.INF_LV / L.LV	0.02 ± 0.01	0.02 ± 0.03	0.02 ± 0.02	0.02 ± 0.01	5.5142	**0.0203**	0.0066	0.4059	6.5787	**0.0114**	0.0076	0.4707

The bold values indicate the *P*-values that were statistically significant, at the threshold of *P* < 0.05.

### 3.4 Volume analyses with region-lateral ventricle proportion

(1) Region-LV proportion for HIV and HAND effects: Another metric we implemented in this study to measure regional atrophy was the region-lateral ventricle proportion, given as the ratio: region_τ_ volume / (region_τ_ volume + Lateral ventricle volume). Using this metric, we detected a larger number of brain regions with patterns of changes associated with HIV pathology. The most pronounced patterns (*p* < 0.00*) were identified in the entire basal ganglia (the pallidum, putamen, and caudate), hippocampus, thalamus, ventral *DC*, third ventricle, fourth ventricle, and inferior lateral ventricle (Table 4). These atrophic patterns were characterized by a greater reduction in LV proportions for the structures of the basal ganglia, hippocampus, thalamus, ventral DC, and inferior LV, and a greater increase in LV proportions for the structures of the third and fourth ventricles. These observations demonstrate that regional atrophy in HIV infection may largely involve the subcortical regions and that the metric accounting for the region proportion of LV may best reveal these hidden patterns of atrophy. We also identified abnormal patterns associated with HAND and non-HAND in these LV-proportion data. These patterns were primarily detected in the hippocampus, thalamus, and pallidum (Supplementary Table S4). These patterns were more pronounced in *HAND*+ subjects (*p* < 0.00*) than they were in *non-HAND* subjects [See for the hippocampus (HAND+, *p* < 0.0089, eff. Size = −0.68386; HAND-, *p* < 0.0812, eff. Size = −0.4453), thalamus (HAND+, *p* < 0.0026, eff. Size = −0.7701; HAND-, *p* < 0.0317, eff. Size = −0.5167), and pallidum (HAND+, *p* < 0.0033, eff. Size = −0.7528; HAND-, *p* < 0.0430, eff. Size = −0.4943)].
(2) Region-LV proportion for age-related effects: In relation to age, our metric of volume proportions identified abnormal patterns, highly pronounced (*p* < 0.000*), in the basal forebrain, caudate nucleus, pallidum, putamen, and hippocampus. Other abnormal patterns (*p* < 0.00*) were detected in the thalamus, ventral *DC*, inferior lateral, and third ventricles.

**Table 4:** Atrophic patterns identified through LV-adjustments via computing regional volume proportions.

Brain structures	HIV	HC	Young	Old	F	*p*	Contrast estimate	Effect size	F	*p*	Contrast estimate	Effect size
Regional volume proportion												
BasalForebrain / (BasalForebrain +LV)	0.06 ± 0.02	0.07 ± 0.02	0.07 ± 0.03	0.06 ± 0.02	12.2823	**0.0006**	0.0137	0.6058	5.5364	**0.0200**	0.0098	0.4318
Caudate / (Caudate +LV)	0.35 ± 0.09	0.38 ± 0.09	0.38 ± 0.09	0.34 ± 0.09	11.898	**0.0007**	0.0516	0.5962	6.6135	**0.0112**	0.0409	0.4719
Hippo / (Hippo +LV)	0.37 ± 0.09	0.41 ± 0.09	0.41 ± 0.09	0.37 ± 0.09	7.6977	**0.0063**	0.0438	0.4796	8.4424	**0.0043**	0.0487	0.5332
Pallidum / (Pallidum +LV)	0.18 ± 0.06	0.2 ± 0.07	0.2 ± 0.06	0.17 ± 0.06	13.4325	**0.0004**	0.0373	0.6335	10.3036	**0.0016**	0.0347	0.5890
Putamen / (Putamen +LV)	0.4 ± 0.1	0.43 ± 0.1	0.44 ± 0.1	0.39 ± 0.1	13.4329	**0.0004**	0.0596	0.6335	7.4447	**0.0072**	0.0471	0.5007
Thalamus / (Thalamus +LV)	0.47 ± 0.1	0.51 ± 0.09	0.51 ± 0.09	0.46 ± 0.1	11.2604	**0.0010**	0.0558	0.5800	11.0371	**0.0011**	0.0587	0.6097
Ventral_DC / (Ventral_DC + LV)	0.4 ± 0.1	0.44 ± 0.09	0.44 ± 0.09	0.4 ± 0.09	11.2572	**0.0010**	0.0529	0.5799	8.7397	**0.0037**	0.0495	0.5425
INF_LV / (INF_LV + LV)	0.03 ± 0.02	0.04 ± 0.04	0.04 ± 0.03	0.03 ± 0.01	6.8970	**0.0096**	0.0106	0.4539	5.8948	**0.0165**	0.0105	0.4455
Third.V / (Third.V + LV)	0.08 ± 0.02	0.09 ± 0.03	0.09 ± 0.02	0.08 ± 0.02	8.2111	**0.0048**	0.0119	0.4953	8.2407	**0.0047**	0.0127	0.5268
Fourth.V / (Fourth.V + LV)	0.11 ± 0.04	0.13 ± 0.05	0.13 ± 0.05	0.11 ± 0.04	5.8097	**0.0172**	0.0179	0.4166	7.1731	**0.0083**	0.0211	0.4915
Ex.CSF / (Ex.CSF + LV)	0.9 ± 0.03	0.91 ± 0.03	0.91 ± 0.03	0.9 ± 0.03	5.6483	**0.0188**	0.0124	0.4108	4.9741	**0.0273**	0.0123	0.4093
R.BasalForebrain / (R.BasalForebrain + R.LV)	0.07 ± 0.03	0.07 ± 0.03	0.08 ± 0.03	0.06 ± 0.02	13.6174	**0.0003**	0.0154	0.6378	5.5448	**0.0199**	0.0105	0.4321
R.Caudate / (R.Caudate + R.LV)	0.36 ± 0.09	0.4 ± 0.09	0.4 ± 0.09	0.35 ± 0.09	12.7648	**0.0005**	0.0546	0.6175	6.8488	**0.0099**	0.0425	0.4802
R.Hippo / (R.Hippo + R.LV)	0.4 ± 0.1	0.44 ± 0.1	0.43 ± 0.1	0.39 ± 0.1	8.5688	**0.0040**	0.0476	0.5060	8.2205	**0.0048**	0.0494	0.5261
R.Pallidum / (R.Pallidum + R.LV)	0.19 ± 0.06	0.21 ± 0.07	0.21 ± 0.07	0.18 ± 0.06	12.4184	**0.0006**	0.0385	0.6091	9.2413	**0.0028**	0.0352	0.5579
R.Putamen / (R.Putamen + R.LV)	0.42 ± 0.1	0.45 ± 0.1	0.46 ± 0.1	0.4 ± 0.1	14.9682	**0.0002**	0.0643	0.6687	7.4412	**0.0072**	0.0481	0.5006
R.Thalamus / (R.Thalamus + R.LV)	0.49 ± 0.1	0.53 ± 0.1	0.53 ± 0.09	0.48 ± 0.11	12.3327	**0.0006**	0.0593	0.6070	10.9869	**0.0012**	0.0595	0.6083
R.Ventral_DC / (R.Ventral_DC + R.LV)	0.42 ± 0.1	0.46 ± 0.1	0.46 ± 0.09	0.41 ± 0.1	12.1973	**0.0006**	0.0566	0.6037	8.4796	**0.0042**	0.0501	0.5344
R.INF_LV / (R.INF_LV + R.LV)	0.04 ± 0.02	0.04 ± 0.04	0.05 ± 0.04	0.04 ± 0.02	5.8863	**0.0165**	0.0114	0.4194	2.6394	0.1065	0.0081	0.2981
L.BasalForebrain / (L.BasalForebrain + L.LV)	0.06 ± 0.02	0.06 ± 0.03	0.06 ± 0.03	0.05 ± 0.02	9.2902	**0.0028**	0.0126	0.5268	5.8323	**0.0170**	0.0106	0.4432
L.Caudate / (L.Caudate + L.LV)	0.33 ± 0.09	0.37 ± 0.09	0.37 ± 0.1	0.33 ± 0.09	9.6467	**0.0023**	0.0488	0.5368	6.7219	**0.0105**	0.0432	0.4758
L.Hippo / (L.Hippo + L.LV)	0.36 ± 0.1	0.4 ± 0.1	0.39 ± 0.1	0.36 ± 0.1	5.8385	**0.0170**	0.0400	0.4177	8.6186	**0.0039**	0.0516	0.5387
L.Pallidum / (L.Pallidum + L.LV)	0.17 ± 0.06	0.2 ± 0.07	0.19 ± 0.07	0.16 ± 0.06	12.4955	**0.0006**	0.0364	0.6110	11.630	**0.0008**	0.0373	0.6258
L.Putamen / (L.Putamen + L.LV)	0.38 ± 0.1	0.42 ± 0.1	0.42 ± 0.1	0.37 ± 0.1	10.3944	**0.0016**	0.0548	0.5573	7.6286	**0.0065**	0.0498	0.5068
L.Thalamus / (L.Thalamus + L.LV)	0.45 ± 0.11	0.5 ± 0.1	0.49 ± 0.1	0.45 ± 0.11	8.8111	**0.0035**	0.0521	0.5131	10.7533	**0.0013**	0.0611	0.6018
L.Ventral_DC / (L.Ventral_DC + L.LV)	0.39 ± 0.1	0.43 ± 0.1	0.43 ± 0.1	0.39 ± 0.1	8.9686	**0.0033**	0.0492	0.5176	9.0438	**0.0031**	0.0525	0.5519
L.INF_LV / (L.INF_LV + L.LV)	0.03 ± 0.01	0.04 ± 0.04	0.04 ± 0.04	0.03 ± 0.01	5.3250	**0.0225**	0.0100	0.3989	7.8321	**0.0059**	0.0128	0.5136

The bold values indicate the *P*-values that were statistically significant, at the threshold of *P* < 0.05.

### 3.5 Receiver operating characteristic analysis

**Figure 1:**
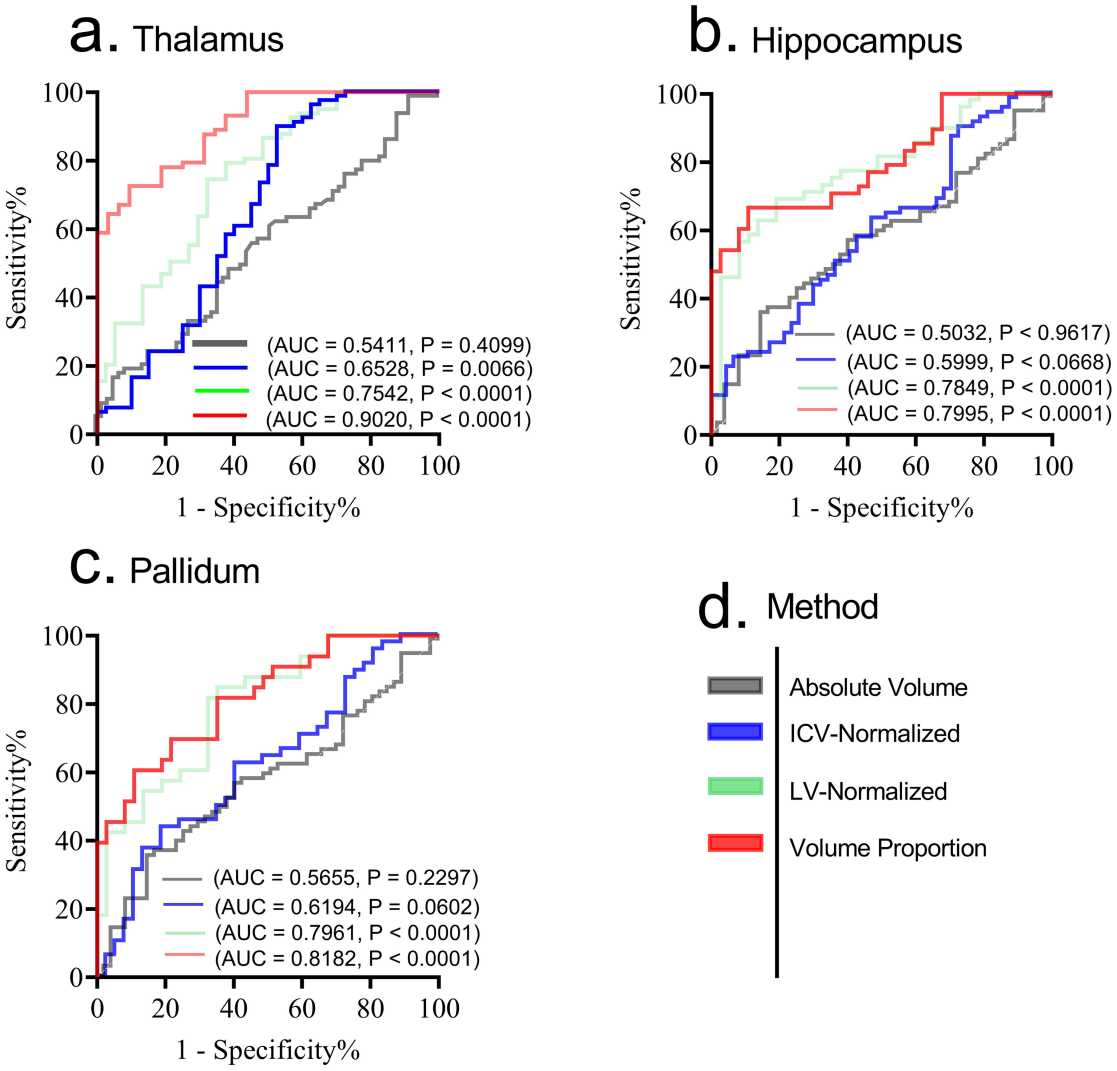
Receiver operating characteristic (ROC) for diagnosing HAND form non-HAND using patterns from thalamus **(a)**, hippocampus **(b)** and pallidum **(c)**. While ICV normalized and LV-normalized data performed relatively better compared to absolute volumes, the strategy that uses volume proportion demonstrated high diagnostic power in distinguishing HAND from non-HAND. **(d)** shows the legend for each method involved. AUC, Area under Curve; *p*, *p*-value. Significance was set at *p* < 0.05.

Given that atrophic patterns associated with HAND+ were mostly identified in the regions of the hippocampus, thalamus, and pallidum, we evaluated the performance of each technique in distinguishing HAND vs. non-HAND using receiver operating characteristic (ROC) analysis. Figure 1 shows the results of this analysis. Briefly, the first approach (using absolute volume) performed poorly in distinguishing HAND from non-HAND. However, ICV-normalized and LV-normalized data performed relatively better. Among these approaches, volume proportion demonstrated high diagnostic power in distinguishing HAND from non-HAND, with AUC values of 0.9020, 0.7995, and 0.8182 for patterns in the thalamus, hippocampus, and pallidum, respectively.

### 3.6 Relationship with clinical measures

**Figure 2:**
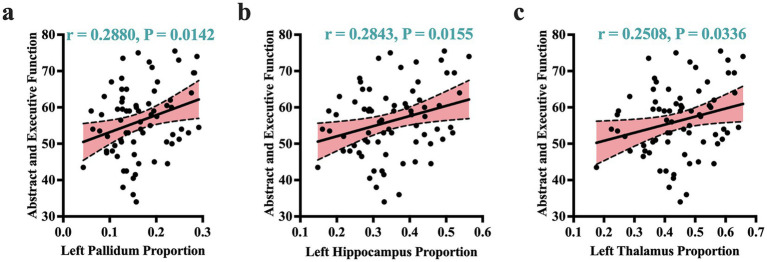
Atrophic correlates of cognitive performance. Atrophic patterns identified by volume proportions correlated significantly with cognitive performance of abstract and executive functions. These patterns of correlations were more dominant in the left pallidum **(a)**, left hippocampus **(b)**, and left thalamus **(c)**. r, Pearson correlation coefficient; *p*, *p*-value. Significance was set at *p* < 0.05.

**Figure 3:**
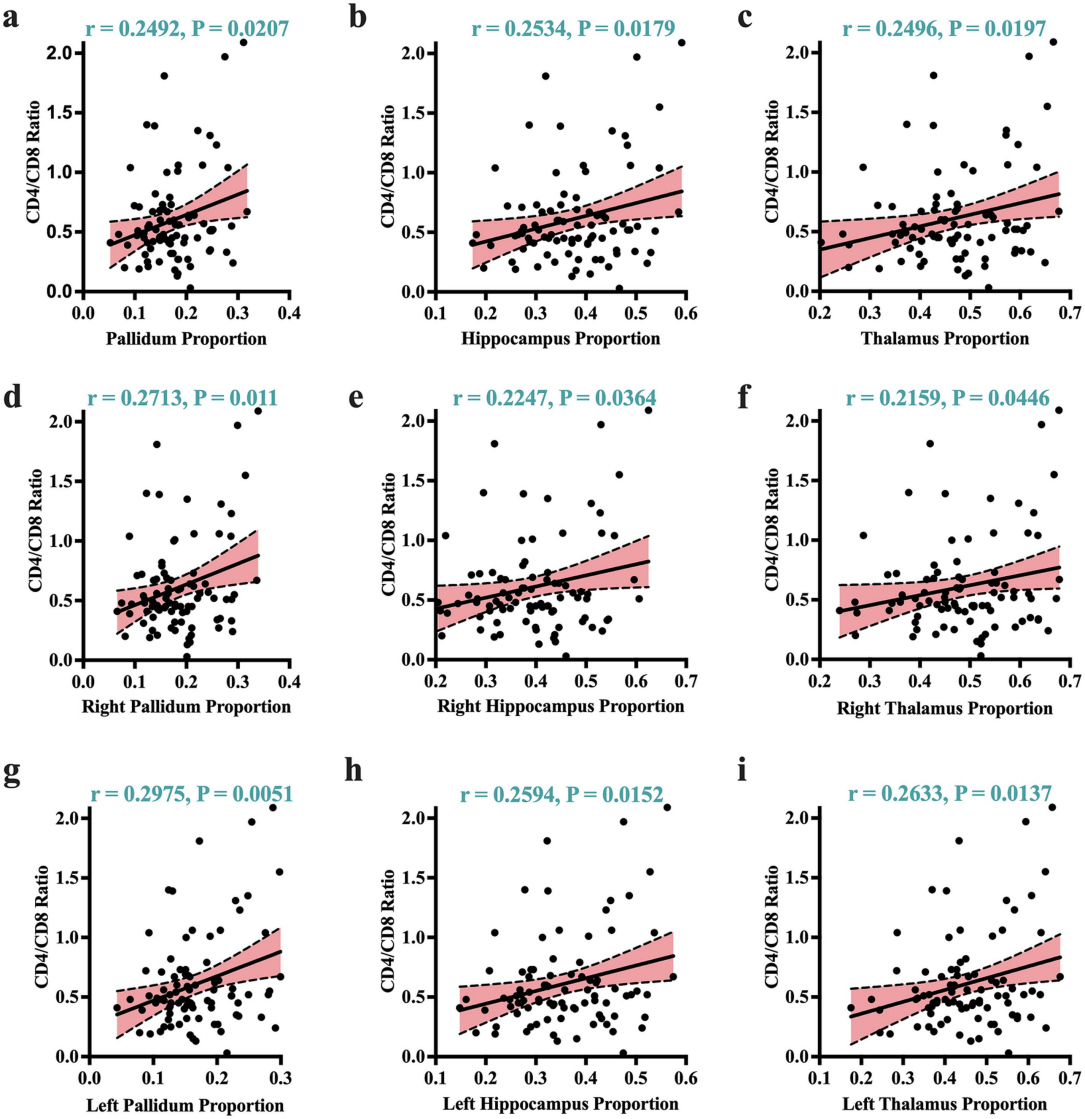
Atrophic correlates of clinical markers. Greater atrophy identified by volume proportions correlated strongly with worsening immunological status (expressed by CD4+/CD8 + ratio). These significant correlates of immunological status were visible across the overall volume proportions of the **(a)** pallidum, **(b)** hippocampus, **(c)** thalamus. They were also visible in the right and left sides of these regions: **(d)** and **(g)** for the right/left pallidum; **(e)** and **(h)** for the right/left hippocampus; and **(f)** and **(i)** for the right/left thalamus. r, Pearson correlation coefficient; *p*, *p*-value. Significance was set at *p* < 0.05.

Given that atrophic patterns associated with *HAND*+ were mostly identified in the regions of the hippocampus, thalamus, and pallidum, we performed correlation analyses to examine the potential relationship between these atrophic patterns and clinical measures. Our analysis revealed that the atrophic patterns in subjects with *HAND* exhibited significant correlations with worsening cognitive scores, especially in the domain of abstract and executive functions (Figure 2; also see Supplementary Table S5). These atrophic patterns also showed strong correlations with the immunological status, particularly the CD4+/CD8 + ratio (Figure 3). No other significant correlations with clinical markers such as the CD4 T-cell count or viral load were reported.

## 4 Discussion

As the core aspect of this study, we utilized different strategies in our analyses to address issues contributing to inconsistencies across studies on reports of structural alterations in HIV and elucidated potential reasons why certain findings may not be reported or acknowledged in other studies. In particular, we investigated the importance of adjusting for both intracranial and ventricular volumes in the analyses of structural changes associated with aging and *HIV*-related conditions. The goal was to establish the significance of controlling individual variability using these two global brain structures in volumetric analyses.

Briefly, upon our analyses, we report that both strategies of normalization and covariation by *ICV* are effective for identifying atrophic patterns associated with either aging or *HIV*. We saw that adjusting for *ICV* through these approaches improved the ability of the structural metrics to detect atrophic patterns in the thalamus (in aging and *HIV*), basal ganglia (in aging and in *HIV*), ventral *DC* (in aging), and lateral ventricle (in aging and *HIV*). Some of these patterns were undetectable when these adjustments were not made. While both covariation and normalization by *ICV* demonstrated a comparable ability to detect atrophic patterns in aging, it is normalization by *ICV*, not covariation by *ICV*, that detected the atrophic patterns in the ventral *DC* in aging, suggesting a slight advantage of this technique over the other. Normalization by *ICV* not only detected *HIV*-associated atrophic patterns across several regions (basal ganglia (pallidum), thalamus, and lateral ventricle) but also proved effective in predicting *HAND* progression, as observed via statistical measures. Interestingly, normalization by *LV* detected more subcortical regions with significant atrophic patterns associated with *HIV* and *HAND*. The atrophic patterns identified by this normalization were pronounced in the hippocampus, basal ganglia (putamen and pallidum), thalamus, ventral DC, third ventricle, and fourth ventricle. The same brain areas, along with additional regions, displayed age-related atrophic patterns with this approach. These areas include the hippocampus, basal ganglia (putamen, pallidum, and caudate nucleus), thalamus, forebrain, and third ventricle.

We also report that a strategy (the fourth strategy) that computes region volume proportions, given by region_τ_ volume / (region_τ_ volume + Lateral ventricle volume), detects the most atrophic patterns related to both aging and *HIV* with more strong statistical strengths. These atrophic patterns revealed by this approach for aging and HIV were more prevalent in the basal ganglia (pallidum, putamen, and caudate nucleus), hippocampus, thalamus, ventral DC, basal forebrain (only in aging), third ventricle, fourth ventricle, and inferior lateral ventricle. Of more importance is that this approach further inform us that the most significant atrophic patterns associated with *HAND* and clinical markers (*CD4/CD8* ratio) were those of the hippocampus, thalamus and pallidum.

**Consistency of observations in basal ganglia and ventricle**: Our results observed in the fourth strategy confirm and are in line with the previous findings reported by Becker et al. (1). The results of their team (1) and ours indicate persisting subcortical brain atrophy, involving the basal ganglia areas of the caudate nucleus and putamen in *HIV*-infected men with well-controlled immune status and viral replication. Previous studies on *HIV* have, also, consistently reported atrophy in the ventricular regions, resulting in increased ventricular volume (spaces) (5, 33). The results from our strategies also validate these previous findings, indicating consistency in ventricular atrophy across studies and strategies. Specifically, we identified atrophic patterns associated with *HIV* in the lateral ventricle (first strategy: raw volumes; second strategy: *ICV* covariation; third strategy: *ICV* normalization), third ventricle (third strategy: LV normalization; fourth strategy: volume proportions), fourth ventricle (third strategy: *LV* normalization; fourth strategy: volume proportions), and inferior lateral ventricle (fourth strategy: volume proportions).

**HIV and aging**: Our findings support the co-occurrence of the effects of both aging and HIV in subjects with *HIV.* This is consistent with reports from other studies (34–36). We also report that despite each factor exerting a distinct influence, they both predominantly target the same regions of the brain. For example, we detected atrophic patterns associated with both aging and *HIV* in the same brain areas of the basal ganglia (putamen, caudate, and pallidum), ventricular areas, thalamus, and hippocampus. However, it is still uncertain whether aging and *HIV* share the same underlying neuropathological processes. Insights from other studies (35) suggest that while *HIV*-specific factors such as immune status and viral load play a significant role in *HIV*-derived brain atrophy, common pathological mechanisms may also be involved in brain atrophy in both HIV and aging. Such mechanisms may include inflammation (37) and *DNA* damage (38–40). Subjects with *HIV* are reported to have increased *DNA* damage, particularly in the mitochondrial *DNA*. It has been demonstrated that subjects with HIV have more pronounced mitochondrial DNA content, mitochondrial DNA deletions, and point mutations than those who are HIV-seronegative (40–43). DNA damage is also a major driver of aging process (44–47). Thus, the elevation of *DNA* damage in subjects with *HIV* accelerates the aging process, potentially causing a faster rate of age-related brain atrophy. Therefore, our study provides corroborating evidence for this phenomenon by reporting more pronounced age-related atrophic patterns in subjects with *HIV* than it would be expected in normal aging. Even in analyses conducted without adjustments for confounding variables, age-related atrophic patterns were more apparent in both the basal ganglia and ventricle areas than those associated with HIV alone. Although we did not provide mechanisms for how HIV interacts with the aging process, these findings indirectly suggest that HIV triggers and accelerates the underlying pathological processes of brain aging.

**Relationship with clinical measures**: A study by Cohen et al. (33) reported a potential association between tissue loss (volume reduction) in the hippocampal and basal ganglia and disease history factors, particularly the nadir CD4 and duration of infection (33). Our study report another dimension of the association between atrophic signatures in the regions of hippocampus, thalamus and pallidum and clinical measures. We observed that the volume proportions (region_τ_ volume / region_τ_ volume + Lateral ventricle volume) of the hippocampus, pallidum, and thalamus, which were significantly reduced in subjects with *HAND*, were strongly associated with lower cognitive performance in abstract and executive functions. These findings suggest that our metric of volume proportion not only distinguishes subjects with *HAND* from normal but can also be used to predict the progression of HIV pathology and serve as a biomarker of dysfunction in the executive domain. Corroborating our findings are the results reported by Lew et al. and Li et al., which demonstrated that HAND is associated with decreases in thalamic and hippocampal gray matte (5, 48).

Specifically, our metric of volume proportion serves as a sensitive marker to distinguish subjects with HIV and HAND from normal subjects and for predicting (through correlation) the progression of HIV pathology, including HAND. This indicates a dual application of this metric, contrary to the FD metric (26). The FD metric seems to be more sensitive to subtle changes in structural complexity, but such changes are more related to cognitive dysfunction and offer less sensitivity to group differences. The usefulness of our metric is comparable to tensor-based morphometry introduced by Chiang et al. (20). This technique visualized brain deficits by comparing two groups through the ratios of Jacobian determinant values and demonstrated that the deficits in HIV subjects were also associated with clinical measures—CD4 + lymphocyte depletion and cognitive impairment. Although the method was completely different, largely relying on fluid image warping and an *α*-entropy-based information-theoretic measure of image correspondence called Jensen-Rényi divergence, its findings in the subcortical areas also included regional atrophy in the putamen, globus pallidus, and thalamus, consistent with our findings.

Similar to many other studies (7, 49), we report that in a well-controlled immune status and viral replication, traditional clinical markers of *HIV*—such as CD4 + count and viral load— may not be primary determinants of *HIV* neuropathological conditions such as *HAND*. We see that these clinical markers did not appear to correlate with any of the neurocognitive performances or neuroimaging structural measures. However, on the other hand, we posit that the *CD4/CD8* ratio may provide deeper insights into the underlying pathology present in the brains of subjects with *HIV*. This assertion is substantiated by our findings, which indeed demonstrated a strong association between a lower *CD4/CD8* ratio and greater atrophy (expressed by volume proportion) in the hippocampus, thalamus, and pallidum. The role of the *CD4/CD8* ratio in understanding the underlying pathogenic conditions such as T-cell pathogenesis (50) and its associations with patterns of brain atrophy (5) or cognitive decline (49) has been discussed previouly. These studies and many others support that the CD4/CD8 ratio may be a sensitive marker for ongoing pathology in cART-treated subjects.

**Limitation**: One limitation worth noting is that controlling for individual variability using these strategies comes with a trade-off. These strategies often overpower the model by obscuring the detection of patterns of differences associated with the variable being controlled for. For example, when *ICV* is controlled, its capacity to reveal its own group differences diminishes, so does the ventricular volume. This occurs because *ICV* itself is effectively normalized to a constant value when normalization is done by *ICV*, thereby nullifying its variability and rendering it incapable of contributing meaningful insights into group-specific disparities. To mitigate this issue, the authors advocate for an independent analysis of these variables before integrating them into the overarching model to control their influence on individual variability in the analyses. By doing this, one can preserve the integrity of the data and the contributions of these variables to the group differences while still accounting for individual variability. Second, we highlight that the time from initial infection or duration on ART can significantly impact brain volume. Unfortunately we did not control for this factor. Therefore, future studies should account for this factor for better inference. Other limitations worth noting are that this is a single-center study and did not account for additional confounders such as opening pressure and increased intracranial pressure (ICP). Meanwhile, it uses a 32 channel head coil which is likely to contribute to decreased SNR compared to 64 head coil. Also, the study is a single case-controlled study with one time point. A cohort prospective study would be more informative.

## 5 Conclusion

In this study, we made attempts to address issues that contribute to a wide range of inconsitencies across studies on structural alterations in *HIV* infection, and elucidate the potential reasons why certain findings may not be reported or acknowledged in other studies.We highlight that different strategies imployed in the individual variability can influence the detection of atrophic patterns asoociated with *HIV* or aging in subjects with *HIV*. We report that while adjusting for *ICV* by normalization or covariation improves the detection of more atrophic patterns that could be overlooked in models without this adjustment, strategies involving the adjustment of *LV* detect most atrophic patterns related to aging and *HIV* across a wide range of brain regions, including the hippocampus, thalamus, and basal ganglia, —and such patterns may better explain *HIV*-associated conditions such as *HAND* and underlying immunological issues often observed in subjects with *HIV* treated with combination antiretroviral therapy. We conclude that models that control for individual variability in intracranial and ventricular volumes have the potential to minimize discrepancies and variations in structural reports of *HIV*, improving the diagnostic power of identified patterns and fostering greater consistency across research studies. These findings contribute to the current body of knowledge, emphasizing the importance of controlling for individual variability when conducting biological analyses to discern patterns associated with neurological conditions.

## Data Availability

The original contributions presented in the study are included in the article/Supplementary material, further inquiries can be directed to the corresponding authors.

## Footnotes

^1^ https://www.volbrain.net/
