# Supplementary material for: Ventricular volume adjustment of brain regions depicts brain changes associated with HIV infection and aging better than intracranial volume adjustment
Source: Front Neurol. 2025 May 19;16:1516168. doi: 10.3389/fneur.2025.1516168 (PMC12127162; doi:10.3389/fneur.2025.1516168)
Supplement: Supplementary file 1 [file Table_1.docx]

|  |  |  |  |  | |  | | |  | | |
| --- | --- | --- | --- | --- | --- | --- | --- | --- | --- | --- | --- |
| **Supplementary Table S1: Atrophic patterns identified from raw volumetric measures and ICV-adjustments through covariation** | | | | | | | | | | | |
|  |  |  |  |  | | **Contrast (HAND-) - HC** | | | **Contrast (HAND+) - HC** | | |
| **Brain structures** | | | **HC** | **HAND-** | **HAND+** | **CE** | ***P*** | **Effect size** | **CE** | ***P*** | **Effect size** |
|  | | |  |  |  |  |  |  |  |  |  |
| **Absolute volumes (mm^3^)** | | |  |  |  |  |  |  |  |  |  |
| BasalForebrain | | | 0.85±0.02 | 0.88±0.01 | 0.91±0.02 | 0.03 | 0.5300 | 0.2707 | 0.0524 | 0.1148 | 0.4729 |
| LV | | | 12.6±0.95 | 15.55±0.81 | 17.03±1.01 | 2.9498 | **0.0500** | 0.4772 | 4.4307 | **0.0056** | 0.7168 |
| R.BasalForebrain | | | 0.43±0.01 | 0.45±0.01 | 0.46±0.01 | 0.0197 | 0.4473 | 0.2892 | 0.0215 | 0.4960 | 0.3153 |
| R.LV | | | 5.95±0.45 | 7.18±0.39 | 7.88±0.48 | 1.2313 | 0.1134 | 0.418 | 1.9333 | **0.0129** | 0.6563 |
| L.BasalForebrain | | | 0.42±0.01 | 0.43±0.01 | 0.45±0.01 | 0.0112 | 0.9874 | 0.1952 | 0.0324 | **0.0401** | 0.5665 |
| L.LV | | | 6.66±0.54 | 8.38±0.46 | 9.15±0.58 | 1.7198 | **0.0483** | 0.4857 | 2.4967 | **0.0066** | 0.7051 |
|  | | |  |  |  |  |  |  |  |  |  |
| **ICV-adjusted volumes by covariation** | | | | |  |  |  |  |  |  |  |
| BasalForebrain | | | 0.86±0.02 | 0.88±0.01 | 0.91±0.02 | 0.0278 | 0.4938 | 0.2785 | 0.0496 | 0.0888 | 0.497 |
| LV | | | 12.64±0.91 | 15.52±0.78 | 16.98±0.97 | 2.8729 | **0.0500** | 0.4823 | 4.3332 | **0.0048** | 0.7274 |
| R.BasalForebrain | | | 0.44±0.01 | 0.45±0.01 | 0.45±0.01 | 0.0183 | 0.3960 | 0.3021 | 0.0197 | 0.4579 | 0.3252 |
| R.LV | | | 5.97±0.43 | 7.16±0.37 | 7.85±0.46 | 1.1905 | 0.1060 | 0.4237 | 1.8816 | **0.0108** | 0.6697 |
| L.BasalForebrain | | | 0.42±0.01 | 0.43±0.01 | 0.45±0.01 | 0.0104 | 1.0000 | 0.1898 | 0.0314 | **0.0371** | 0.5732 |
| L.LV | | | 6.67±0.53 | 8.36±0.45 | 9.12±0.56 | 1.6837 | **0.0477** | 0.4867 | 2.4509 | **0.0063** | 0.7085 |
