# Supplementary material for: Ventricular volume adjustment of brain regions depicts brain changes associated with HIV infection and aging better than intracranial volume adjustment
Source: Front Neurol. 2025 May 19;16:1516168. doi: 10.3389/fneur.2025.1516168 (PMC12127162; doi:10.3389/fneur.2025.1516168)
Supplement: Supplementary file 2 [file Table_2.docx]

|  |  |  |  |  |  |  |  |  |  |  |  |
| --- | --- | --- | --- | --- | --- | --- | --- | --- | --- | --- | --- |
| **Supplementary Table S2: Atrophic patterns identified through ICV-adjustments via normalization** | | | | | | | | | | | |
|  |  |  |  |  |  | **Contrast (HAND-) - HC** | | | **Contrast (HAND+) - HC** | | |
| **Brain structures** | | | **HC** | **HAND-** | **HAND+** | **CE** | ***P*** | **Effect size** | **CE** | ***P*** | **Effect size** |
| **IC fraction** | | |  |  |  |  |  |  |  |  |  |
| Thalamus normalized | | | 0.0093±0.00009 | 0.0089±0.00008 | 0.0089±0.0001 | -0.0004 | **0.0101** | -0.5945 | -0.0004 | **0.0071** | -0.7001 |
| LV normalized | | | 0.0087±0.00062 | 0.0106±0.00053 | 0.0116±0.00066 | 0.0019 | 0.0544 | 0.4766 | 0.0029 | **0.0054** | 0.7201 |
| R.Thalamus normalized | | | 0.0047±0.00005 | 0.0045±0.00004 | 0.0044±0.00005 | -0.0002 | **0.0111** | -0.5884 | -0.0003 | **0.0033** | -0.7526 |
| R.LV normalized | | | 0.0041±0.00029 | 0.0049±0.00025 | 0.0054±0.00031 | 0.0008 | 0.1116 | 0.4193 | 0.0013 | **0.0133** | 0.6538 |
| L.Thalamus normalized | | | 0.0046±0.00005 | 0.0045±0.00004 | 0.0044±0.00005 | -0.0002 | **0.0219** | -0.5427 | -0.0002 | **0.0354** | -0.5769 |
| L.LV normalized | | | 0.0046±0.00036 | 0.0057±0.0003 | 0.0062±0.00038 | 0.0011 | 0.0514 | 0.481 | 0.0017 | **0.0063** | 0.7092 |
