# Supplementary material for: Ventricular volume adjustment of brain regions depicts brain changes associated with HIV infection and aging better than intracranial volume adjustment
Source: Front Neurol. 2025 May 19;16:1516168. doi: 10.3389/fneur.2025.1516168 (PMC12127162; doi:10.3389/fneur.2025.1516168)
Supplement: Supplementary file 3 [file Table_3.docx]

|  |  |  |  |  |  |  |  |  |  |  |  |
| --- | --- | --- | --- | --- | --- | --- | --- | --- | --- | --- | --- |
| **Supplementary Table S3: Atrophic patterns identified through LV-adjustments via normalization** | | | | | | | | | | | |
|  |  |  |  |  |  | **Contrast (HAND-) - HC** | | | **Contrast (HAND+) - HC** | | |
| **Brain structures** | | | **HC** | **HAND-** | **HAND+** | **CE** | ***P*** | **Effect size** | **CE** | ***P*** | **Effect size** |
| **LV fraction** | | |  |  |  |  |  |  |  |  |  |
| BasalForebrain / LV | | | 0.08±0.004 | 0.07±0.003 | 0.06±0.004 | -0.0094 | 0.2253 | -0.3575 | -0.0143 | 0.0531 | -0.5426 |
| Caudate / LV | | | 0.67±0.035 | 0.59±0.03 | 0.53±0.037 | -0.0777 | 0.2752 | -0.3384 | -0.1434 | **0.0196** | -0.6242 |
| Hippo / LV | | | 0.78±0.042 | 0.67±0.036 | 0.6±0.045 | -0.1169 | 0.1051 | -0.4243 | -0.1863 | **0.0099** | -0.6761 |
| Pallidum / LV | | | 0.28±0.015 | 0.23±0.012 | 0.2±0.015 | -0.0471 | **0.0409** | -0.498 | -0.0721 | **0.0029** | -0.7614 |
| Putamen / LV | | | 0.86±0.047 | 0.75±0.04 | 0.65±0.05 | -0.1155 | 0.1792 | -0.3784 | -0.2078 | **0.0093** | -0.6806 |
| Thalamus / LV | | | 1.19±0.062 | 1±0.053 | 0.89±0.066 | -0.196 | **0.0490** | -0.4845 | -0.3062 | **0.0031** | -0.7569 |
| Ventral_DC / LV | | | 0.89±0.047 | 0.76±0.04 | 0.67±0.05 | -0.1333 | 0.0897 | -0.4373 | -0.2156 | **0.0064** | -0.7075 |
| INF_LV / LV | | | 0.05±0.004 | 0.04±0.004 | 0.03±0.005 | -0.0117 | 0.1403 | -0.3999 | -0.0154 | 0.0636 | -0.5269 |
| Third.V / LV | | | 0.11±0.004 | 0.09±0.004 | 0.09±0.005 | -0.0153 | **0.0282** | -0.5251 | -0.0165 | **0.0396** | -0.5676 |
| Fourth.V / LV | | | 0.16±0.009 | 0.13±0.008 | 0.12±0.009 | -0.023 | 0.1374 | -0.4017 | -0.039 | **0.0094** | -0.6797 |
| R.BasalForebrain / R.LV | | | 0.04±0.002 | 0.03±0.002 | 0.03±0.002 | -0.0043 | 0.2861 | -0.3347 | -0.0074 | **0.0391** | -0.5686 |
| R.Caudate / R.LV | | | 0.34±0.018 | 0.3±0.015 | 0.26±0.019 | -0.0399 | 0.2482 | -0.3484 | -0.0717 | **0.0191** | -0.6264 |
| R.Hippo / R.LV | | | 0.4±0.021 | 0.34±0.018 | 0.3±0.023 | -0.0601 | 0.0960 | -0.4318 | -0.0968 | **0.0075** | -0.6958 |
| R.Pallidum / R.LV | | | 0.14±0.007 | 0.11±0.006 | 0.1±0.008 | -0.0229 | 0.0558 | -0.4747 | -0.0351 | **0.0048** | -0.7283 |
| R.Putamen / R.LV | | | 0.43±0.023 | 0.37±0.02 | 0.33±0.025 | -0.0585 | 0.1645 | -0.386 | -0.1055 | **0.0076** | -0.6954 |
| R.Thalamus / R.LV | | | 0.6±0.031 | 0.5±0.026 | 0.44±0.033 | -0.099 | **0.0429** | -0.4944 | -0.1577 | **0.002** | -0.7877 |
| R.Ventral_DC / R.LV | | | 0.44±0.023 | 0.38±0.02 | 0.33±0.025 | -0.0663 | 0.0885 | -0.4384 | -0.1079 | **0.0059** | -0.7137 |
| R.INF_LV / R.LV | | | 0.02±0.002 | 0.02±0.002 | 0.02±0.003 | -0.0044 | 0.4889 | -0.2796 | -0.0071 | 0.1491 | -0.4475 |
| L.BasalForebrain / L.LV | | | 0.04±0.002 | 0.03±0.002 | 0.03±0.002 | -0.0051 | 0.2088 | -0.3645 | -0.0068 | 0.0937 | -0.4920 |
| L.Caudate / L.LV | | | 0.33±0.018 | 0.3±0.015 | 0.26±0.019 | -0.0379 | 0.3081 | -0.3274 | -0.0717 | **0.0206** | -0.6204 |
| L.Hippo / L.LV | | | 0.38±0.021 | 0.33±0.018 | 0.29±0.022 | -0.0569 | 0.1157 | -0.4163 | -0.0895 | **0.0132** | -0.6546 |
| L.Pallidum / L.LV | | | 0.14±0.007 | 0.12±0.006 | 0.1±0.008 | -0.0242 | **0.0316** | -0.5168 | -0.0371 | **0.0019** | -0.7919 |
| L.Putamen / L.LV | | | 0.43±0.024 | 0.37±0.02 | 0.33±0.025 | -0.0572 | 0.1919 | -0.3722 | -0.1024 | **0.0113** | -0.6660 |
| L.Thalamus / L.LV | | | 0.59±0.031 | 0.5±0.027 | 0.45±0.033 | -0.0971 | 0.0569 | -0.4732 | -0.1486 | **0.005** | -0.7244 |
| L.Ventral_DC / L.LV | | | 0.45±0.024 | 0.38±0.02 | 0.34±0.025 | -0.0668 | 0.0928 | -0.4345 | -0.1077 | **0.0071** | -0.7000 |
| L.INF_LV / L.LV | | | 0.02±0.002 | 0.02±0.002 | 0.02±0.003 | -0.0072 | 0.0800 | -0.4465 | -0.0082 | 0.079 | -0.5076 |
| ALPS_L / LV | | | 0.13±0.007 | 0.11±0.006 | 0.09±0.007 | -0.0261 | **0.0128** | -0.5793 | -0.0436 | **0.0001** | -0.9664 |
| ALPS_R / LV | | | 0.13±0.007 | 0.11±0.006 | 0.09±0.007 | -0.0216 | **0.0479** | -0.4862 | -0.0395 | **0.0004** | -0.8879 |
| ALPS_B / LV | | | 0.13±0.007 | 0.11±0.006 | 0.09±0.007 | -0.0234 | **0.0264** | -0.5298 | -0.0411 | **0.0002** | -0.9292 |
| ALPS_L_CSF / LV | | | 0.01±0.0005 | 0.01±0.0004 | 0.01±0.0005 | -0.0015 | 0.0639 | -0.4642 | -0.0025 | **0.0021** | -0.7836 |
| ALPS_R_CSF / LV | | | 0.01±0.0005 | 0.01±0.0004 | 0.01±0.0005 | -0.0011 | 0.2425 | -0.3505 | -0.0021 | **0.0069** | -0.7024 |
| ALPS_B_CSF / LV | | | 0.01±0.0005 | 0.01±0.0004 | 0.01±0.0005 | -0.0012 | 0.1278 | -0.4079 | -0.0023 | **0.0033** | -0.7530 |
