# Supplementary material for: Ventricular volume adjustment of brain regions depicts brain changes associated with HIV infection and aging better than intracranial volume adjustment
Source: Front Neurol. 2025 May 19;16:1516168. doi: 10.3389/fneur.2025.1516168 (PMC12127162; doi:10.3389/fneur.2025.1516168)
Supplement: Supplementary file 4 [file Table_4.docx]

| **Supplementary Table S4: Atrophic patterns identified through computing regional volume proportions** | | | | | | | | | | | |
| --- | --- | --- | --- | --- | --- | --- | --- | --- | --- | --- | --- |
|  |  |  |  |  |  | **Contrast (HAND-) - HC** | | | **Contrast (HAND+) - HC** | | |
| **Brain structures** | | | **HC** | **HAND-** | **HAND+** | **CE** | ***P*** | **Effect size** | **CE** | ***P*** | **Effect size** |
| **Proportion** | | |  |  |  |  |  |  |  |  |  |
| BasalForebrain / (BasalForebrain +LV) | | | 0.07±0.003 | 0.06±0.003 | 0.06±0.004 | -0.0082 | 0.2115 | -0.3633 | -0.0124 | 0.0505 | -0.5470 |
| Caudate / (Caudate +LV) | | | 0.39±0.013 | 0.36±0.011 | 0.34±0.014 | -0.0333 | 0.1659 | -0.3853 | -0.0537 | **0.0207** | -0.6202 |
| Hippo / (Hippo +LV) | | | 0.42±0.014 | 0.38±0.012 | 0.36±0.015 | -0.0406 | 0.0812 | -0.4453 | -0.0624 | **0.0089** | -0.6839 |
| Pallidum / (Pallidum +LV) | | | 0.21±0.009 | 0.18±0.008 | 0.17±0.01 | -0.0291 | **0.0430** | -0.4943 | -0.0443 | **0.0033** | -0.7528 |
| Putamen / (Putamen +LV) | | | 0.45±0.014 | 0.41±0.012 | 0.38±0.015 | -0.0375 | 0.1397 | -0.4003 | -0.0633 | **0.0101** | -0.6746 |
| Thalamus / (Thalamus +LV) | | | 0.53±0.015 | 0.48±0.013 | 0.45±0.016 | -0.0496 | **0.0317** | -0.5167 | -0.0740 | **0.0026** | -0.7701 |
| Ventral_DC / (Ventral_DC +LV) | | | 0.45±0.014 | 0.41±0.012 | 0.39±0.015 | -0.0411 | 0.0759 | -0.4507 | -0.0639 | **0.0071** | -0.7004 |
| INF_LV / (INF_LV +LV) | | | 0.04±0.004 | 0.03±0.003 | 0.03±0.004 | -0.0093 | 0.1468 | -0.3960 | -0.0124 | 0.0635 | -0.5271 |
| Third.V / (Third.V +LV) | | | 0.09±0.004 | 0.08±0.003 | 0.08±0.004 | -0.0123 | **0.0339** | -0.5118 | -0.0132 | 0.0503 | -0.5472 |
| Fourth.V / (Fourth.V +LV) | | | 0.13±0.007 | 0.12±0.006 | 0.1±0.007 | -0.0167 | 0.1579 | -0.3896 | -0.0286 | **0.0110** | -0.6680 |
| Ex.CSF / (Ex.CSF +LV) | | | 0.91±0.005 | 0.9±0.004 | 0.9±0.005 | -0.0099 | 0.3024 | -0.3293 | -0.0164 | 0.0515 | -0.5453 |
| R.BasalForebrain / (R.BasalForebrain + R.LV) | | | 0.08±0.004 | 0.07±0.003 | 0.06±0.004 | -0.0086 | 0.2289 | -0.3560 | -0.0136 | **0.0427** | -0.5612 |
| R.Caudate / (R.Caudate + R.LV) | | | 0.41±0.014 | 0.37±0.012 | 0.35±0.014 | -0.0356 | 0.1368 | -0.4021 | -0.0542 | **0.0226** | -0.6129 |
| R.Hippo / (R.Hippo + R.LV) | | | 0.45±0.014 | 0.41±0.012 | 0.38±0.015 | -0.0415 | 0.0850 | -0.4416 | -0.0630 | **0.0107** | -0.6705 |
| R.Pallidum / (R.Pallidum + R.LV) | | | 0.22±0.01 | 0.19±0.008 | 0.18±0.01 | -0.0300 | 0.0560 | -0.4744 | -0.0442 | **0.0071** | -0.7003 |
| R.Putamen / (R.Putamen + R.LV) | | | 0.47±0.015 | 0.43±0.013 | 0.4±0.016 | -0.0387 | 0.1341 | -0.4038 | -0.0641 | **0.0111** | -0.6677 |
| R.Thalamus / (R.Thalamus + R.LV) | | | 0.55±0.015 | 0.5±0.013 | 0.47±0.016 | -0.0503 | **0.0322** | -0.5154 | -0.0750 | **0.0026** | -0.7685 |
| R.Ventral_DC / (R.Ventral_DC + R.LV) | | | 0.47±0.014 | 0.43±0.012 | 0.41±0.015 | -0.0420 | 0.0782 | -0.4484 | -0.0638 | **0.0092** | -0.6815 |
| R.INF_LV / (R.INF_LV + R.LV) | | | 0.05±0.004 | 0.04±0.004 | 0.03±0.004 | -0.0064 | 0.7209 | -0.2351 | -0.0110 | 0.2279 | -0.4042 |
| L.BasalForebrain / (L.BasalForebrain + L.LV) | | | 0.07±0.004 | 0.06±0.003 | 0.05±0.004 | -0.0093 | 0.1601 | -0.3884 | -0.0128 | 0.0587 | -0.5339 |
| L.Caudate / (L.Caudate + L.LV) | | | 0.38±0.014 | 0.34±0.012 | 0.32±0.015 | -0.0352 | 0.1614 | -0.3877 | -0.0569 | **0.0190** | -0.6266 |
| L.Hippo / (L.Hippo + L.LV) | | | 0.41±0.015 | 0.36±0.013 | 0.34±0.016 | -0.0436 | 0.0717 | -0.4552 | -0.0652 | **0.0092** | -0.6813 |
| L.Pallidum / (L.Pallidum + L.LV) | | | 0.21±0.009 | 0.17±0.008 | 0.16±0.01 | -0.0313 | **0.027**5 | -0.5267 | -0.0474 | **0.0017** | -0.7980 |
| L.Putamen / (L.Putamen + L.LV) | | | 0.43±0.015 | 0.39±0.013 | 0.37±0.016 | -0.0403 | 0.1240 | -0.4105 | -0.066 | **0.0103** | -0.6732 |
| L.Thalamus / (L.Thalamus + L.LV) | | | 0.51±0.016 | 0.46±0.013 | 0.44±0.017 | -0.0523 | **0.0321** | -0.5156 | -0.076 | **0.0035** | -0.7493 |
| L.Ventral_DC / (L.Ventral_DC + L.LV) | | | 0.44±0.015 | 0.4±0.012 | 0.38±0.015 | -0.0438 | **0.0664** | -0.4612 | -0.0672 | **0.0064** | -0.7077 |
| L.INF_LV / (L.INF_LV + L.LV) | | | 0.04±0.004 | 0.03±0.003 | 0.03±0.004 | -0.0122 | **0.0474** | -0.4870 | -0.0139 | **0.0464** | -0.5542 |
